# Short-Term Temporal Metabolic Behavior in Halophilic Cyanobacterium *Synechococcus* sp. Strain PCC 7002 after Salt Shock

**DOI:** 10.3390/metabo9120297

**Published:** 2019-12-05

**Authors:** Shimpei Aikawa, Atsumi Nishida, Tomohisa Hasunuma, Jo-Shu Chang, Akihiko Kondo

**Affiliations:** 1 Graduate School of Science, Technology, and Innovation, Kobe University, 1-1 Rokkodai, Nada-ku, Kobe 657-8501, Japan; saikawa@affrc.go.jp (S.A.); akondo@kobe-u.ac.jp (A.K.); 2 Graduate School of Engineering, Kobe University, 1-1 Rokkodai, Nada-ku, Kobe 657-8501, Japan; nnn.nxa23.atm@gmail.com; 3 Engineering Biology Research Center, Kobe University, 1-1 Rokkodai, Nada-ku, Kobe 657-8501, Japan; 4 Department of Chemical Engineering, National Cheng Kung University, Tainan 701, Taiwan; changjs@mail.ncku.edu.tw; 5 Research Center for Energy Technology and Strategy, National Cheng Kung University, Tainan 701, Taiwan; 6 Center for Bioscience and Biotechnology, National Cheng Kung University, Tainan 701, Taiwan; 7 Biomass Engineering Program, RIKEN, 1-7-22 Suehiro, Tsurumi-ku, Yokohama 230-0045, Japan

**Keywords:** salt stress, cyanobacteria, compatible solute, Na^+^/H^+^ antiporter, K^+^ uptake, polyamine, metabolomics

## Abstract

In response to salt stress, cyanobacteria increases the gene expression of Na^+^/H^+^ antiporter and K^+^ uptake system proteins and subsequently accumulate compatible solutes. However, alterations in the concentrations of metabolic intermediates functionally related to the early stage of the salt stress response have not been investigated. The halophilic cyanobacterium *Synechococcus* sp. PCC 7002 was subjected to salt shock with 0.5 and 1 M NaCl, then we performed metabolomics analysis by capillary electrophoresis/mass spectrometry (CE/MS) and gas chromatography/mass spectrometry (GC/MS) after cultivation for 1, 3, 10, and 24 h. Gene expression profiling using a microarray after 1 h of salt shock was also conducted. We observed suppression of the Calvin cycle and activation of glycolysis at both NaCl concentrations. However, there were several differences in the metabolic changes after salt shock following exposure to 0.5 M and 1 M NaCl: (i): the main compatible solute, glucosylglycerol, accumulated quickly at 0.5 M NaCl after 1 h but increased gradually for 10 h at 1 M NaCl; (ii) the oxidative pentose phosphate pathway and the tricarboxylic acid cycle were activated at 0.5 M NaCl; and (iii) the multi-functional compound spermidine greatly accumulated at 1 M NaCl. Our results show that *Synechococcus* sp. PCC 7002 acclimated to different levels of salt through a salt stress response involving the activation of different metabolic pathways.

## 1. Introduction

Cyanobacteria are oxygenic photosynthetic prokaryotes that hold promise for producing sustainable fuels and chemicals because of their fast growth compared to higher plants. In addition, some halophilic cyanobacteria can grow under high salinity conditions that are incompatible with plant growth [1,2]. Advances in our understanding of the salt stress response in halophilic cyanobacteria would lead to enhanced salt tolerance of photosynthetic organisms, including cyanobacteria, microalgae, and higher plants. For example, heterogonous expression of genes encoding compatible solute synthesis enzymes or Na^+^/H^+^ antiporters from the halophilic cyanobacteria *Aphanothece halophytica* increases the salt tolerance of the freshwater cyanobacterium *Synechococcus* sp. PCC 7942 and of *Arabidopsis thaliana*, a higher plant [3,4]. In particular, the introduction of higher salt-resistance into cyanobacteria and microalgae will allow their cultivation in highly concentrated seawater, thus preventing reduced productivity because of contamination by other microorganisms [5].

Salt stress response has been studied in several cyanobacterial species such as *Synechocystis* sp. and *Synechococcus* sp. [2]. During this response, cyanobacterial cells (i) shrink rapidly because of water efflux immediately following NaCl addition [6]; (ii) Na^+^ and Cl^−^ ions flow into the cytoplasm [7]; (iii) toxic sodium-ions in the cytoplasm are exchanged by external potassium-ions via Na^+^/H^+^ antiporters and K^+^ uptake systems [8]; and (iv) compatible solutes accumulate and increase the intracellular pressure [9]. Cyanobacteria acclimate to higher salt concentrations via these responses over a period of 12–24 h.

Cyanobacteria have multiple genes encoding Na^+^/H^+^ antiporters. For example, there are six different genes (*nhaS1-S6*) annotated as Na^+^/H^+^ antiporters in *Synechocystis* sp. PCC 6803. NhaS3 is mainly localized to the thylakoid membrane and is essential for cell viability even in low-sodium medium [10]. In addition, disruption of both the *nhaS4* and *nhaS5* genes has no effect on the salt tolerance [11]. The salt tolerance of the *nhaS2* deletion mutant is unchanged but the gene is required in low-salt medium for ion homeostasis [12]. Although the functions of some Na^+^/H^+^ antiporters are poorly understood, several Na^+^/H^+^ antiporters are believed to play a role in sodium-ion export from the cytoplasm in the salt stress response of cyanobacteria (e.g., NhaS1 and/or NhaS6 in the case of *Synechocystis* sp. PCC 6803).

K^+^ uptake in the salt stress response of cyanobacteria generally involves a sodium ion-dependent Ktr system (composed of the three subunits KtrA, KtrB, and KtrE) and an ATP dependent Kdp system (composed of the five subunits KdpA, KdpB, KdpC, KdpD, and KdpF) [2]. The Kdp system in *Escherichia coli* is responsible for the immediate uptake of K^+^ after salt shock. However, a mutant of *Synechocystis* sp. PCC 6803 in which the Kdp system subunit gene *kdpA* is deleted can grow in 0.8 M NaCl, whereas a mutant lacking the Ktr system subunit gene *ktrB* is sensitive to 0.1 M NaCl [13]. Accordingly, the Kdp system is believed to be of minor importance in the cyanobacterial salt stress response [2,13].

Compatible solutes in cyanobacteria differ depending on the salt-tolerance of the strain [2]. Freshwater strains with low halotolerance, such as *Nostoc muscorum*, accumulate sucrose and/or trehalose as their major compatible solute. Moderately halophilic strains, such as *Synechocystis* sp. PCC 6803 and *Synechococcus* sp. PCC 7002, are characterized by glucosylglycerol as their main compatible solute, whereas halophilic strains such as *A. halophytica* usually synthesize glycine betaine or glutamate betaine [2].

In the salt stress response process following the addition of 0.68 M NaCl, transcriptomic analysis using microarrays revealed that *Synechocystis* sp. PCC 6803 upregulates 360 genes and downregulates 200 genes [14,15]. Approximately half of all genes in *Synechocystis* sp. PCC 6803 encode unknown functional proteins [15], but salt stress induces genes related to Na^+^/H^+^ antiporters (*nhaS1, nhaS3, nhaS6*), the potassium-uptake system subunit (*ktrB*), and genes related to glucosylglycerol biosynthesis (*ggpS*, *glpD, glpK,* and *stpA*) [14]. Glucosylglycerol synthesis is related to photosynthetic carbon fixation and glycogen degradation [16,17,18,19,20]. However, there has been little research regarding the roles of other metabolic pathways such as glycolysis, the Calvin cycle, the oxidative pentose phosphate (OPP) pathway, and the tricarboxylic acid cycle (TCA) cycle. In particular, there have been no studies on changes in intermediate metabolite concentrations during the short-term (24 h) salt stress response of cyanobacteria.

We conducted metabolite profiling of the halophilic cyanobacterium *Synechococcus* sp. PCC 7002 from 1 to 24 h after salt shock induced by transferring the cells from freshwater medium (0 M NaCl) to high-salinity medium containing 0.5 or 1 M NaCl. Profiling was performed using capillary electrophoresis/mass spectrometry (CE/MS) and gas chromatography/mass spectrometry (GC/MS). We also analyzed the expression levels of multiple genes after 1 h of salt shock using a microarray to support our metabolomic analysis data.

## 2. Results and Discussion

### 2.1. Cell Growth after Transfer to 0.5 and 1 M NaCl Conditions

**Figure 1:**
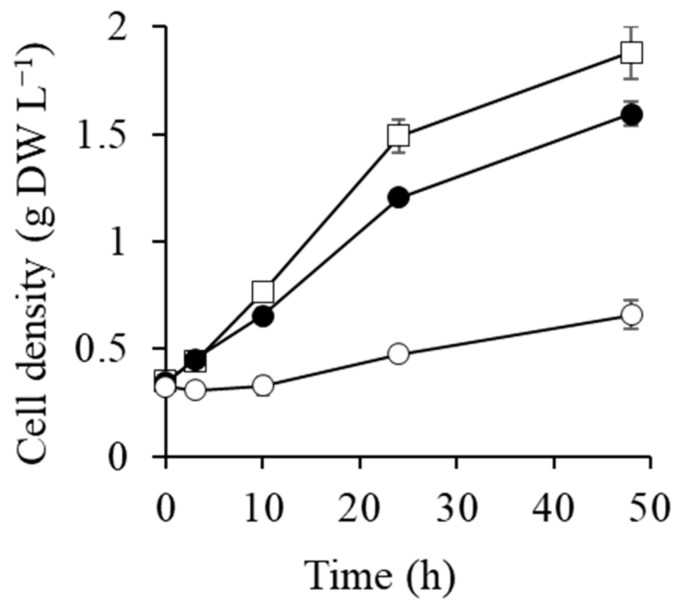
Growth curves for *Synechococcus* sp. PCC 7002 in different media without NaCl addition (control; open squares), with 0.5 M NaCl (filled circles), and with 1 M NaCl (open circles). Error bars indicate standard deviations (SD) of three replicate experiments. For some data points, the error bars obtained using three replicates are smaller than the symbols.

Cell growth curves after transfer to the different media are shown in Figure 1. Cell growth in 0.5 M NaCl was slightly slower than in the control, with the cell density of the control and 0.5 M NaCl cultures being 0.76 and 0.65 g dry-cell weight (DW) L^−1^ at 10 h, respectively. The cell growth stopped until 10 h upon suddenly increasing the salt concentration to 1 M from 0 M NaCl (Pre-cultured condition), and then they grew again from 10 h (Figure 1). The cells have probably acclimated to 1 M NaCl condition after 10 h.

### 2.2. Gene Expression of Na^+^/H^+^ Antiporters and K^+^ Transporters

*Synechococcus* sp. PCC 7002 possesses six different genes annotated as Na^+^/H^+^ antiporters, identified from the genome on the National Center for Biotechnology Information website (https://www.ncbi.nlm.nih.gov). The gene expression level of *nhaS3* (A0577) increased 2.4-fold under the 1 M NaCl condition compared with the control (Table 1). Marin et al. reported the transient induction of several Na^+^/H^+^ antiporter genes (*nhaS1*, *nhaS3*, and *nhaS6*) in *Synechocystis* sp. PCC 6803 after the addition of 684 mM NaCl [14]. Since NhaS3 is mainly localized in the thylakoid membrane in *Synechocystis* sp. PCC 6803 [10], NhaS1 and NhaS6 are predicted to play a role in the exocytic release of Na^+^ from the cytoplasm. However, we did not observe two-fold higher induction of other Na^+^/H^+^ antiporter genes (*nhaS1*, *nhaS2*, *nhaS4*, *nhaS5*, and *nhaS6*) under both NaCl conditions. Another candidate Na^+^ transport system in the cytoplasmic membrane of *Synechococcus* sp. PCC 7002 is the F_1_F_0_-type Na^+^-ATPase homologous operon (*atpA-II, atpB-II, atpC-II, atpD*(A0749), *atpF-II, atpG-II, and atpH-II*), which may be absent in the *Synechocystis* sp. PCC 6803 genome [21]. The F_1_F_0_-type Na^+^-ATPase has the broad ion specificity for Na^+^, H^+^, or Li^+^ in an anaerobic bacterium *Propionigenium modestum* [22,23]. The expression of these genes was enhanced in *Synechococcus* sp. PCC 7002 after 2 h of salt shock at 0.5 M and 1 M NaCl (Table 1). However, such gene expression changes have not observed after acclimating to hyper saline condition in *Synechococcus* sp. PCC 7002 [24]. Accordingly, F_1_F_0_-type Na^+^-ATPase are likely induced for short-term after salt shock in *Synechococcus* sp. PCC 7002. F_1_F_0_-type Na^+^-ATPase in cyanobacteria is generally believed to be an ATP synthase that uses the ΔNa^+^ between the inside and the outside of the cell [21]. However, when driven in reverse, Na^+^-ATPase performs as an efficient sodium pump in *P. modestum* [25]. Na^+^-ATPase may play two roles in *Synechococcus* sp. PCC 7002: as an ATP synthase, and as a sodium pump in the salt stress response.

The gene expression level of *ktrB*, a Ktr system subunit, is enhanced three-fold within 1 h of NaCl salt shock in *Synechocystis* sp. PCC 6803, while that of *kdpA*, a Kdp system subunit, does not change [7]. The Kdp system was previously reported to be of minor importance in the cyanobacterial salt stress response [2,20]. However, the gene expression levels of the Kdp system subunits were enhanced by salt shock in *Synechococcus* sp. PCC 7002 (Table 1), while those of the Ktr system subunits did not change. The expression levels of *kdpA, kdpB, kdpC, kdpD*, and *kdpF* was increased 32.0-, 33.0-, 13.9-, 20.2-, and 20.6-fold at 0.5 M NaCl, respectively (Table 1), although the gene expression of Ktr system was less than two-fold changes. Further investigations are needed to clarify the role of the Kdp system in the salt stress response of cyanobacteria. However, to our knowledge, this is the first report of the Kdp system responding to NaCl stress in cyanobacteria. *Synechococcus* sp. PCC 7002 is a good model strain for studying the cyanobacterial Kdp system.

### 2.3. Metabolic Analysis under 0.5 M and 1 M NaCl Conditions

#### 2.3.1. Compatible Solute Synthesis

**Figure 2:**
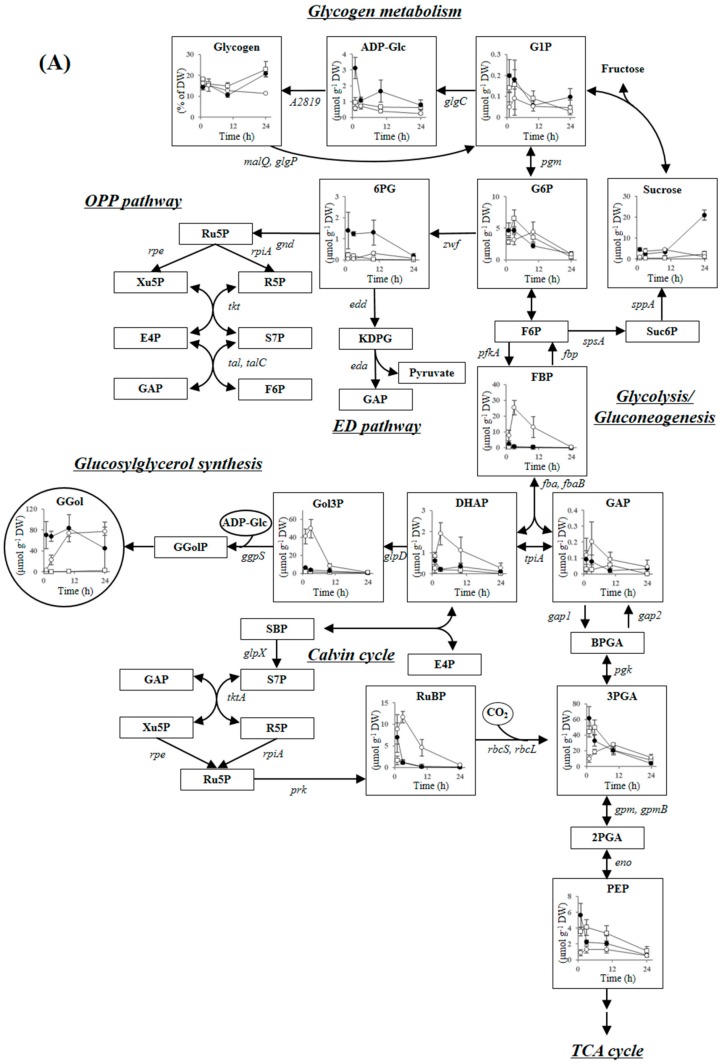

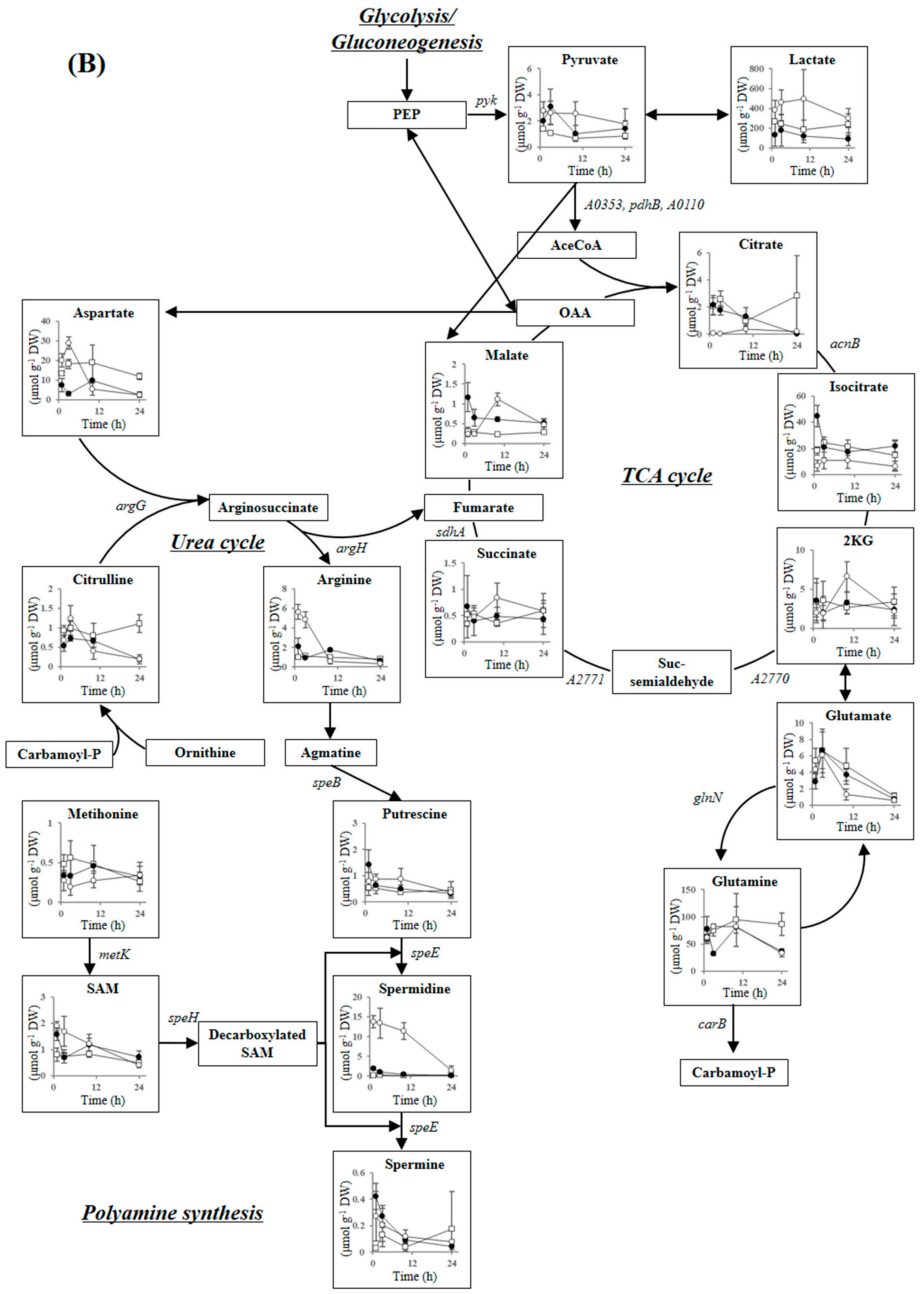
Metabolite concentrations in (**A**) glycogen metabolism, the OPP pathway, the ED pathway, glucosylglycerol synthesis, the Calvin cycle, and glycolysis/gluconeogenesis, and (**B**) the TCA cycle, the urea cycle, and polyamine synthesis of *Synechococcus* sp. PCC 7002 cells cultivated in different media: without NaCl (control; open squares), with 0.5 M NaCl (filled circles), and with 1 M NaCl (open circles). The gene names given in Table 2, Table 3, Table 4 and Table 5 are shown for each reaction. Error bars indicate SD of three replicate experiments. Abbreviations: AceCoA, acetyl-CoA; ADP-Glc, ADP-glucose; BPGA, 1,3-bisphosphoglycerate; Carbamoyl-P, carbamoyl phosphate; DHAP, dihydroxyacetone phosphate; E4P, erythrose-4-phosphate; FBP, fructose-1,6-bisphosphate; F6P, fructose-6-phosphate; GAP, glyceraldehyde-3-phosphate; G1P, glucose-1-phosphate; G6P, glucose-6-phosphate; GGol, glucosylglycerol; GGolP, glucosylglycerol 3-phosphate; Gol3P, glycerol 3-phosphate; KDPG, 2-keto-3-deoxy-6-phosphogluconate; 2KG, 2-ketoglutarate; OAA, oxaloacetate; PEP, phosphoenolpyruvate; 6PG, 6-phosphogluconate; 2PGA, 2-phosphoglycerate; 3PGA, 3-phosphoglycerate; R5P, ribose-5-phosphate; Ru5P, ribulose-5-phosphate; RuBP, ribulose-1,5-bisphosphate; S7P, sedoheptulose-7-phosphate; SAM, S-adenosylmethionine; SBP, sedoheptulose-1,7-bisphosphate; Suc, succinate; Suc6P, Sucrose-6-phosphate; Xu5P, xylulose-5-phosphate.

The moderately halotolerant cyanobacterium *Synechococcus* sp. PCC 7002 accumulates glucosylglycerol as the major compatible solute and sucrose as a secondary compatible solute [26]. Figure 2 shows the changes under different NaCl conditions in metabolite concentrations in glucosylglycerol synthesis, glycogen metabolism, glycolysis/gluconeogenesis, the Entner–Doudoroff (ED) pathway, the Calvin cycle, the OPP pathway, the TCA cycle, the urea cycle, and polyamine synthesis. Glucosylglycerol accumulated much faster at 0.5 M than 1 M NaCl. The glucosylglycerol level at 0.5 M NaCl was 70 μmol g^−1^ DW after 1 h and 84 μmol g^−1^ DW after 10 h. In contrast, the glucosylglycerol level at 1 M NaCl was just 3 μmol g^−1^ DW after 1 h, gradually increasing to 74 μmol g^−1^ DW after 10 h. Glucosylglycerol is synthesized from ADP-glucose and glycerol 3-phosphate via glucosylglycerol-phosphate by the cooperation of glycerol-3-phosphate dehydrogenase (GlpD), glycerol-phosphate synthase (GgpS) and glucosylglycerol-phosphate phosphatase (Figure 2). The GlpD and GgpS genes were highly induced at both NaCl conditions (Table 2). However, glucosylglycerol accumulation was much slower at 1 M than 0.5 M NaCl. Glycerol 3-phosphate, the precursor metabolite of glucosylglycerol, accumulated after 3 h only at 1 M NaCl (50 μmol g^−1^ DW), which is more than 20-fold of that observed at 0.5 M NaCl. In addition, the concentration of ADP-glucose was up to 1 μmol g^−1^ DW at 1 M NaCl after 1-24 h, which was much lower than that obtained at 0.5 M NaCl. These results show that glucosylglycerol synthesis might be inhibited by a deficiency of ADP-glucose at 1 M NaCl.

Sucrose is believed a secondary compatible solute in *Synechococcus* sp. PCC 7002 because its concentration is much lower than that of glucosylglycerol. Sucrose accumulated at both NaCl conditions after 1 h, to a maximum concentration of around 1 μmol g^−1^ DW at 1 M NaCl (Figure 2). The gene expression of sucrose-phosphate synthase (SpsA) and sucrose-phosphate phosphatase (SppA) were enhanced at both 0.5 M and 1 M NaCl (Table 2). Sucrose level increased to 21 μmol g^−1^ DW at 0.5 M NaCl after 24 h (Figure 2). Similar increase of sucrose is observed in *Synechococcus* sp. PCC 7002 after acclimating to hyper saline condition [27]. Sucrose might play a role for long-term salt response in cyanobacteria.

#### 2.3.2. Glycogen Metabolism and ADP-Glucose Synthesis

Cyanobacteria accumulate glycogen as a carbon sink during photosynthesis and consume glycogen as a carbon source to control intracellular metabolic homeostasis. The glycogen content fluctuates because of the changes in metabolic balance in response to cultivation conditions such as light intensity, CO_2_ concentration, and nutrient concentration [28,29]. Glycogen is likely interconverted to glucosylglycerol by salt stress in some cyanobacteria [18,20], including *Synechococcus* sp. PCC 7002 [19]. A decrease in glycogen at 0.5 M and 1 M NaCl was inversely correlated with an increase in glucosylglycerol in this study (Figure 2). The glycogen content decreased to 14.2% of DW after 1 h at 0.5 M NaCl compared to the control, and decreased to 12.7% of DW after 10 h at 1 M NaCl. There was no significant difference in glycogen metabolism gene expression between 0.5 M and 1 M NaCl. The gene expressions levels of alpha-glucan phosphorylase (GlgP) and 4-alpha-glucanotransferase (MalQ), which are related to glycogen degradation, were enhanced around two-fold at both conditions. The gene expression of glycerol-3-phosphate dehydrogenase (GlgC), a regulatory enzyme for glycogen production [30], and 1,4-alpha-glucan branching enzyme (A2819), were suppressed by around half at both conditions (Table 2). Glycogen degradation was probably enhanced by salt stress at both NaCl conditions.

**Figure 3:**
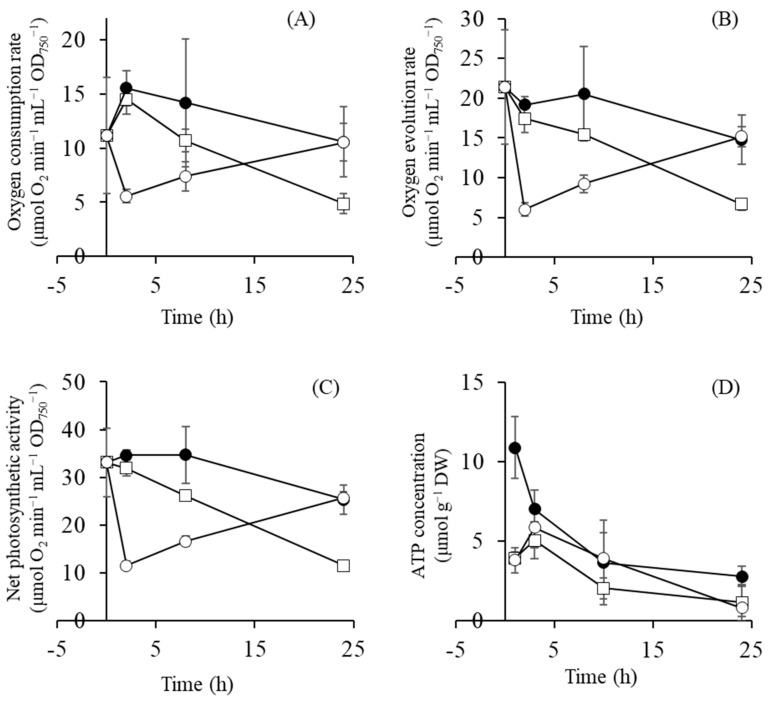
Oxygen consumption rate (**A**), oxygen evolution rate (**B**), net photosynthetic activity (**C**), and ATP concentration (**D**) in *Synechococcus* sp. PCC 7002 cells cultivated in different media: without NaCl (control; open squares), with 0.5 M NaCl (filled circles), and with 1 M NaCl (open circles). Error bars indicate SD of three replicate experiments.

ADP-glucose synthesis by GlgC enzyme is dependent on the ATP concentration. The oxygen consumption rate, oxygen evolution rate, and net photosynthetic activity related to ATP production under the control, 0.5 M and 1 M NaCl conditions are shown in Figure 3, in addition to the ATP concentrations. The oxygen consumption rate, oxygen evolution rate, and net photosynthetic activity after 2 h at 0.5 M NaCl were similar to the control. However, the oxygen consumption rate and oxygen evolution rate decreased to one-third and one-fourth after 2 h at 1 M NaCl. Net photosynthetic activity, calculated from the sum of the oxygen consumption rate and the oxygen evolution rate, decreased to one-third after 2 h at 1 M NaCl. The ATP concentration was much lower (around half) after 1 h at 1 M NaCl than at 0.5 M NaCl (Figure 3). ADP-glucose synthesis might be suppressed at 1 M NaCl because of a decrease in photosynthetic ATP-production, resulting in the delay of glucosylglycerol synthesis at 1 M NaCl as shown in Figure 2.

#### 2.3.3. Carbon Dioxide Fixation, Glycolysis, the ED Pathway and the OPP Pathway

Ribulose-1,5-bisphosphate (RuBP) carboxylase/oxygenase (Rubisco) is inactivated in the presence of a high concentration of NaCl in both halophytes and glycophytes [1,31]. In this study, RuBP accumulated 3–4-fold higher at both NaCl conditions than in the control after 1 h (Figure 2), indicating that Rubisco in *Synechococcus* sp. PCC 7002 is inhibited by a sudden increase in NaCl concentration, similar to higher plants. Transcriptomic analysis showed that expression of the Rubisco large subunit gene (*rbcL*), Rubisco small subunit gene (*rbcS*), and Rubisco chaperone gene (*rbcX*) were greatly decreased at both NaCl conditions (Table 3). The genes encoding NADP(H)-dependent glyceraldehyde-3-phosphate dehydrogenase (Gapdh2) and phosphoribulokinase (Prk), which are believed to regulate the Calvin cycle by altering the ratio of NADP(H)/NAD(H) [32,33], were downregulated at both NaCl conditions. In addition, the fructose-1,6-bisphosphatase/sedoheptulose-1,7-bisphosphatase gene (*glpX*) and the CO_2_ concentrating mechanism (CCM) genes *ccmK*, *ccmL*, and *ccmM* were also downregulated at both conditions (Table 3). These results show that *Synechococcus* sp. PCC 7002 may suppress gene expression levels related to carbon dioxide fixation, along with inactivation of the Rubisco enzyme. CcmO is a predominant protein of β-carboxysome shell, and the CCM activity of the *ccmO* deletion mutant is significantly decreased [34].The gene expression level of *ccmO* in *Synechococcus* sp. PCC 7002 does not increase after acclimating to 1.5 M NaCl condition [24]. However, CcmO gene was up-regulated 2.4-fold after 0.5 M NaCl salt shock, while that was not changed at 1 M NaCl (Table 3). CcmO might play a role for maintaining β-carboxysome activity under moderately salt stress condition, although further investigations are needed to clarify the mechanism.

Each value indicates the ratio of the level expression in stressed cells to that in control cells. Values shown are means (±standard deviations) of three independent experiments.

The concentrations of fructose-1,6-bisphosphate (FBP), dihydroxyacetone phosphate (DHAP), and glyceraldehyde-3-phosphate (GAP) increased after 1 h at 0.5 M and 1 M NaCl compared to the control. The concentrations of FBP, DHAP, and GAP were 2.5, 0.6, and 0.1 μmol g^−1^ DW at 0.5 M NaCl, and those were 7.9, 0.9, and 0.1 μmol g^−1^ DW at 1 M NaCl (Figure 2). Gene expression in the unidirectional glycolysis enzymes phosphofructokinase (PfkA) and NAD(H)-dependent glycelaldehyde-3-phosphate dehydrogenase (Gapdh1, a key enzyme in regulating carbon flow in glycolysis [35]) were induced 2–3-fold at both NaCl conditions (Table 3). The gene expression of fructose-1,6-bisphosphate aldolase class I (FbaB) was also increased 2–3-fold at both NaCl conditions (Table 3). In contrast, the gene expression of glycolytic enzymes such as phosphoglycerate kinase, phosphoglycerate mutase, and 2-phosphopyruvate hydratase, which convert GAP to pyruvate, were suppressed at both NaCl conditions (Table 3). Glycolysis reactions may be partially activated between glucose 1-phosphate (G1P) and GAP or DHAP to concentrate precursor metabolites (FBP, DHAP, and GAP) for glucosylglycerol production.

The concentration of FBP, DHAP, and GAP increased further after 3 h at 1 M NaCl, but decreased to the same level as the control at 0.5 M NaCl (Figure 2). Glycolysis must remain active even after 3 and 10 h at 1 M NaCl because the glucosylglycerol concentration was insufficient for establishing adequate intracellular pressure.

The ED pathway is also important for sugar catabolism of cyanobacteria in addition to glycolysis and the OPP pathway [36]. The gene expression of 6-phosphogluconate dehydratase was suppressed at both NaCl conditions (Table 3). In contrast, 2-Keto-3-deoxygluconate-6-phosphate aldolase gene was upregulated 3.5-fold at both NaCl conditions. The ED pathway might be suppressed after salt shock of 0.5 M and 1 M NaCl by the downregulation of 6-phosphogluconate dehydratase gene.

The OPP pathway metabolite 6-phosphogluconate (6PG) greatly accumulated after 1-10 h at 0.5 M NaCl compared to the control (Figure 2). The genes encoding the OPP pathway enzymes glucose-6-phosphate 1-dehydrogenase and fructose-1,6-bisphosphatase (Fbp) were upregulated at 0.5 M NaCl (Table 3). The reactive oxygen species are more generated by salt-stress in cyanobacteria, which are detoxified via NADPH-dependent enzymes such as catalase, ascorbate peroxidase, and glutathione reductase [37]. The OPP pathway generate NADPH and are also involved in the production of a precursor metabolite for glucosylglycerol synthesis. The OPP pathway is probably activated for producing NADPH and glucosylglycerol under salt-stressed condition. However, the OPP pathway was less activated at 1 M NaCl than at 0.5 M NaCl. The 6PG concentration at 1 M NaCl was lower than at 0.5 M NaCl after 1-10 h (Figure 2). The gene expression levels of *zwf* and *fbp* were lower at 1 M than 0.5 M NaCl (Table 3). Glycolysis may be activated in preference to NADPH production via the OPP pathway at 1 M NaCl.

#### 2.3.4. Tricarboxylic Acid Cycle, Urea Cycle, and Polyamine Synthesis

Isocitrate and malate, the substrates for reducing NAD cofactor in the TCA cycle, accumulated temporarily after 1 h at 0.5 M NaCl, then decreased after 3 h (Figure 2). The gene expression levels of 2-ketoglutarate decarboxylase (A2770), succinate-semialdehyde dehydrogenase (A2771), and succinate dehydrogenase subunit (SdhA) were upregulated 2-3-fold at 0.5 M NaCl (Table 4). The TCA cycle is likely activated to produce more reducing agent for salt stress response, similar to the OPP pathway. In addition, succinate dehydrogenase may act as the main respiratory donor [38]. Energization of both the thylakoid and cytoplasmic membrane electron transfer chains is important under salt stress conditions in cyanobacteria [39], possibly to provide a proton gradient for Na^+^/H^+^ transporter and ATP production to supplement photosynthetic activity. *Synechococcus* sp. PCC 7002 may obtain protons and electrons from succinate, in addition to the photosynthetic oxidation of water at 0.5 M NaCl.

Citrate and isocitrate levels were lower at 1 M NaCl than in the control after 1-10 h (Figure 2). The gene expression of pyruvate dehydrogenase (A0353, A0655, A0110) were decreased at both NaCl conditions (Table 4). However, since the pyruvate dehydrogenase is a salt sensitive enzyme in wheat plant *Triticum aestivum* [40], the pdh dehydrogenase activity at 1 M NaCl may be suppressed more than that at 0.5 M NaCl. In addition, the gene expression of methylisocitrate dehydratase (AcnB) was suppressed less at 1 M NaCl than at 0.5 M NaCl, and gene expression levels of A2771 were induced less at 1 M NaCl than at 0.5 M NaCl (Table 4). In contrast, metabolites related to the urea cycle, including arginine and aspartate, accumulated at 1 M NaCl after 1 and 3 h. Arginine and aspartate are the precursor metabolites of polyamines, and thus polyamine synthesis may be activated in preference to the production of reducing agents via the TCA cycle at 1 M NaCl.

Polyamines are polycationic aliphatic amines found in all living organisms and affect DNA folding, protein synthesis, membrane stability, photosystem II stability, and stress responses, although the underlying mechanisms are not completely understood [41]. Intracellular polyamine level increases because of salt stress in both halophytes and glycophytes [42,43]. Some polyamines, including putrescine, spermine, and spermidine, are found in cyanobacteria. An exogenous addition of spermidine enhanced the cell growth of *Synechocystis* sp. PCC 6803 in the presence of 0.5 M NaCl [44]. As shown in Figure 2, spermidine concentrations increased to 2.0 and 13.8 μmol g^−1^ DW at 0.5 M NaCl and 1 M NaCl after 1 h. The spermidine concentration remained high at 1 M NaCl (above 10 μmol g^−1^ DW) but decreased gradually at 0.5 M NaCl (Figure 2). The spermine concentration was much lower than that of spermidine but increased at both NaCl conditions after 1 h. The concentration of putrescine remained essentially constant under all conditions. Spermidine is the major polyamine accumulating in *Synechococcus* sp. PCC 7002 under salt stress conditions. Short-term increases in polyamines are observed in various higher plants such as a narrow-leafed ash *Fraxinus angustifolia* and a pepper *Capsicum annuum* [45,46], but such increases have not been investigated in cyanobacteria. Since polyamines accumulate transiently also in the presence of 0.2 M mannitol in *F. angustifolia* [45], polyamine is likely also related to osmotic response in addition to ionic adjustment. We believe that polyamines aid short-term salt tolerance in cyanobacteria also.

The spermidine concentration increased at both NaCl conditions after 1 h. However, the expression levels of genes related to polyamine synthesis (*speB, speE, metK,* and *speH*) and the urea cycle (*argG* and *argH*) significantly decreased at both 0.5 M and 1 M NaCl after 1 h (Table 4). The expression levels of genes related to polyamine synthesis might be enhanced in the very short term, then suppressed within several hours. Our results show that polyamines, especially spermidine, are probably important in the early stage of the salt stress response in *Synechococcus* sp. PCC 7002.

Each value indicates the ratio of the level expression in stressed cells to that in control cells. Values shown are means (±standard deviations) of three independent experiments.

### 2.4. Gene Expression of Photosynthetic Components

Transcriptomic analysis revealed down-regulation of a large number of genes encoding subunits of photosystem I (PSI), subunits of photosystem II (PSII), phycobilisome (PBS), cytochrome *b_6_f* complex, F type H^+^-transporting ATP synthase, and electron carrier proteins (ferredoxin, ferredoxin-NADP reductase, and cytochrome *c_6_*) at both 0.5 M and 1 M NaCl conditions. Several of the genes involved in the photosynthetic apparatus are listed in Table 5 and others are listed in Supplementary Materials Table S1.

In contrast, the *A2164* gene (which encodes PsbA) was up-regulated at both conditions, and the genes encoding phycocyanobilin lyase CpcE and CpcF were up-regulated at 1 M NaCl. Two main isoforms of protein PsbA, D1:1 and D1:2, are found in some cyanobacteria such as *Synechocystis* sp. PCC 6803 and *Synechococcus* sp. PCC 7942 [47]. The isoform D1:1 is more abundant in *Synechococcus* sp. PCC 7942 under optimal growth conditions, whereas D1:2 is upregulated when the cells are exposed to stress conditions such as high illumination or UVB levels [47]. The A0157 gene and the A2164 gene are upregulated, and the A1418 gene is downregulated in *Synechococcus* sp. PCC 7002 after acclimating to hyper saline condition [24]. We could presume that the D1:1 isoform is A0157 and A2164 and the D1:2 isoform is A1418. However, in contrast to long-term response, the A0157 gene was downregulated at short-term response (Table 5). Accordingly, there is likely also different function between A0157 and A2164 in *Synechococcus* sp. PCC 7002.

Each value indicates the ratio of the level expression in stressed cells to that in control cells. Values shown are means (±standard deviations) of three independent experiments.

### 2.5. Concluding Remarks on Salt Stress Response of Synechococcus sp. PCC 7002 at 0.5 M and 1 M NaCl

**Figure 4:**
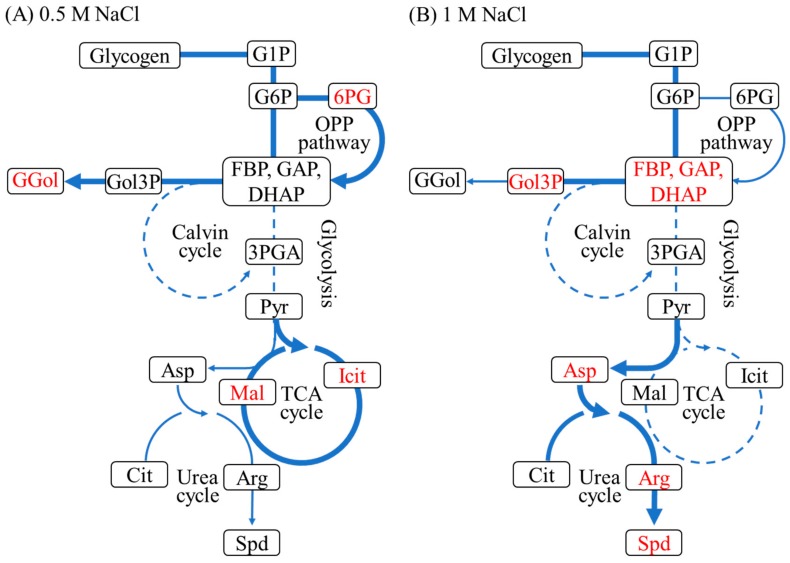
Alterations in metabolism at 0.5 M NaCl (**A**) and 1 M NaCl (**B**) after 1–3 h. Activated, slightly activated, and suppressed metabolic pathways are shown as bold solid lines, thin solid lines, and dotted lines, respectively. Accumulated metabolites are shown in red.

Figure 4 shows activated and suppressed metabolic pathways at 0.5 M and 1 M NaCl after 1–3 h of salt shock. Glycogen degradation and glycolysis were activated at both NaCl conditions and the Calvin cycle was suppressed. However, there were also differences between cells exposed to 0.5 M and 1 M NaCl. The TCA cycle and the OPP pathway, accompanied by the production of reducing agents, were activated at 0.5 M NaCl, whereas high levels of the cationic polyamine spermidine accumulated at 1 M NaCl. These differences are probably due to the rate of glucosylglycerol synthesis. The level of glucosylglycerol at 1 M NaCl after 10 h was the same as at 0.5 M NaCl at 1 h (Figure 2). The delay of glucosylglycerol synthesis at 1 M NaCl was likely due to a decrease in photosynthetic ATP-production (Figure 3). Furthermore, the slow synthesis of glucosylglycerol at 1 M NaCl prolonged the inhibition of cell growth, photosynthetic activity, and Rubisco enzyme activity because of the high intracellular Na^+^ level. Correspondingly, as the glucosylglycerol concentration increased at 1 M NaCl, photosynthesis activity and Rubisco enzyme activity recovered gradually, and cell growth resumed after 10 h (Figure 1, Figure 2 and Figure 3). These results show that rapid glucosylglycerol accumulation is among the most important responses by *Synechococcus* sp. PCC 7002 for acclimating to higher salt conditions. However, even with high intracellular Na^+^ levels, *Synechococcus* sp. PCC 7002 continued photosynthesis and produced glucosylglycerol slowly, indicating that it endures high salt stress by accumulating spermidine, thereby maintaining a suitable cation–anion balance and stabilizing membranes. *Synechococcus* sp. PCC 7002 thus overcomes metabolic inhibition because of salt stress by activating different metabolic pathways, depending on the salt stress level.

## 3. Materials and Methods

### 3.1. Strain and Culture Medium

*Synechococcus* sp. PCC 7002 was obtained from the Pasteur Culture Collection (Paris, France). Cells were pre-cultured in hexagonal flat flasks containing 600 mL fresh modified A medium without NaCl (AF medium) under continuous illumination at 400 µmol photons m^−2^ s^−1^ at 32 °C and bubbling air containing 2% (*v/v*) CO_2_ at 100 mL min^−1^. AF medium contained 1.5 g L^−1^ NaNO_3_, 40 mg L^−1^ K_2_HPO_4_, 75 mg L^−1^ MgSO_4_•7H_2_O, 36 mg L^−1^ CaCl_2_•2H_2_O, 31.5 mg L^−1^ Na_2_EDTA•2H_2_O, 8.0 mg L^−1^ FeCl_3_•6H_2_O, 34.3 mg L^−1^ H_3_BO_3_, 4.3 mg L^−1^ MnCl_2_•4H_2_O, 0.315 mg L^−1^, ZnCl_2_, 30 μg L^−1^ Na_2_MoO_4_•2H_2_O, 3.0 μg L^−1^ CuSO_4_•5H_2_O, 12.2 μg L^−1^, CoCl_2_•6H_2_O, 4.0 μg L^−1^ cobalamin, and 8.3 mM Tris aminomethane (pH 8.2). All reagents were purchased from Nacalai Tesque, Inc., (Kyoto, Japan). After pre-culturing in medium without NaCl for 24 h, the cells were inoculated into three type of medium without added NaCl (defined as control), containing 0.5 M NaCl, and containing 1 M NaCl at 0.32 g DW L^−1^, and then cultivated at the same culturing conditions as the pre-culture for 48 h.

### 3.2. Cell Density

Cell growth was monitored turbidimetrically using the optical density at 750 nm (OD_750_) measured using a UVmini-1240 (Shimadzu, Kyoto, Japan). Cell concentration was calculated as DW in medium from the linear correlation between DW and OD_750_. We determined OD_750_ of 1 that corresponds to approximately 0.33 g DW L^−1^.

### 3.3. Extraction of Intercellular Metabolites

*Synechococcus* sp. PCC 7002 cells, equivalent to 5 mg DW, were collected from the cultivation media and injected at a ratio of 4:1 (*v/v*) into a solution of 32.5% (*v/v*) methanol in water pre-chilled at −30 °C to rapidly terminate metabolism. The cells were harvested by centrifugation at 15,000× *g* for 5 min using a centrifuge and rotor precooled at −20 °C. The cells were resuspended in 2 mL methanol containing 190 nM (+)-10-camphorsulfonic acid, 31 μM *L*-methionine sulfone, and 31 μM piperazine-1,4-*bis*(2-ethanesulfonic acid) (PIPES) as internal standards for mass analysis. Intracellular metabolites were extracted with a cold mixture of methanol-chloroform-water (10:3:1, by vol.), as described previously [48]. The mixture was shaken at 1200 rpm in a Bioshaker MBR-022UP (TAITEC, Saitama, Japan) for 30 min at 4 °C in the dark, then centrifuged at 22,000× *g* for 5 min at 4 °C. The supernatant cell extract (980 μL) was transferred to a clean tube, 440 μL MilliQ water was added, and the aqueous and organic layers were separated by centrifugation at 22,000× *g* for 5 min at 4 °C. Two aliquots (450 μL each) of the aqueous layer were transferred to clean tubes for analysis by CE/MS and GC/MS. After removing the solubilized proteins using a 5 kDa cut-off filter (Millipore, Bedford, MA, USA), the extracts were evaporated under reduced pressure using a Plus freeze dry system (FreeZone 2.5, Labconco, Kansas, MO, USA). The dried extracts were stored at –80 °C until used for metabolite analysis.

### 3.4. Metabolite Analysis Using CE/MS

The dried extracts were dissolved in 20 μL Milli-Q water and analyzed using an Agilent G7100 CE system, an Agilent G6224AA LC/MSD time-of-flight (TOF) system, and an Agilent 1200 series isocratic high-performance liquid chromatography (HPLC) pump equipped with a 1:100 splitter for delivery of the sheath liquid (Agilent Technologies, Palo Alto, CA, USA). Agilent Chem-Station software for CE/MS and MassHunter software for TOFMS were used as the system control and data acquisition software. The CE/MS analytical conditions for cationic and anionic metabolite analyses were as described previously [49].

### 3.5. Glucosylglycerol and Sucrose Quantification Using GC/MS

Dried extracts were derivatized as described previously [50]. The derivatives (1 μL) were analyzed by GC/MS (GCMSQP-2010 system, Shimadzu) using the following conditions: column, CP-Sil 8 CB low bleed (30 m × 0.25 mm i.d. DF 0.25 μm; Varian Inc., Palo Alto, CA, USA); carrier gas, helium; flow rate, 1 mL min^−1^ at constant flow; injector temperature, 230 °C; transfer line, 250 °C; ion source, 200 °C; injection volume, 1 μL; injection mode, split (1:25); oven temperature, 80 °C for 2 min, ramped to 230 °C at a rate of 15 °C min^−1^, 230 °C for 6 min; solvent delay, 3.5 min; electron voltage: −70 eV; and detection mode, SIM at *m/z*: 73 and 204. The peak areas of glucosylglycerol and sucrose were determined using GCMS Solution ver. 4.0 (Shimadzu).

### 3.6. Glycogen Quantification

Glycogen was extracted from cells as described previously [49]. Cells (10 mg DW) in 200 μL KOH (30%, w/v) were incubated in a heat block for 90 min at 95 °C and then placed on ice. The glycogen was precipitated by adding 600 μL ethanol pre-chilled to 4 °C to each cell extract and keeping the samples on ice for 1 h. The extracts were centrifuged at 3000× *g* for 5 min at 4 °C, then the pellets were washed twice with cold ethanol and dried for 10 min at 60 °C in a heat block. Each dried sample was reconstituted in 100 μL water, centrifuged at 10,000× *g* for 5 min at 4 °C, and the supernatant was subjected to HPLC analysis as described previously [51].

### 3.7. Measurement of Oxygen Consumption Rate and Oxygen Evolution Rate

The oxygen consumption and evolution rates were measured at 32 °C with a Clark-type oxygen electrode DW2/2 (Hansatech Instruments Ltd., King’s Lynn, UK) controlled by a computerized oxygen monitoring system (OMS, Hansatech Instruments Ltd.). Oxygen consumption was monitored for 2 min under dark conditions, then oxygen evolution was monitored for 3 min by irradiation at 400 μmol photons m^−2^ s^−1^ using a halogen light source. The cell concentration in the reaction mixture was adjusted to 1.0 OD_750_ for each cultivation medium. The net photosynthesis rate was calculated from the sum of the oxygen consumption rate and the oxygen evolution rate.

### 3.8. Profiles of Transcriptional Activity

Gene expression profiles of *Synechococcus* sp. PCC 7002 cultivated under different NaCl conditions were analyzed using a custom 8 × 15K microarray for 3187 *Synechococcus* sp. PCC 7002 genes (Agilent Technologies). After 1 h cultivation, the cells were harvested by centrifugation at 22,000× *g* for 5 min at 4 °C and immediately frozen in liquid nitrogen. Total RNA was extracted using a Total RNA Isolation Mini kit (Agilent Technologies) and complementary DNA labeled with cyanine 3 was generated using a Low Input Quick Amp Labeling kit (Agilent Technologies) according to the manufacture’s protocol. After incubation at 65 °C for 17 h, the slides were scanned using an Agilent DNA Microarray scanner (Agilent Technologies). The scanning data were quantified and analyzed using Agilent feature extraction software and GeneSpring GX software (Agilent Technologies). We calculated fold-changes from the transcript level under 0.5 M and 1 M NaCl relative to control condition, and set fold-change threshold to more than 2.

## Figures and Tables

**Table 1:** Gene expression of Na^+^/H^+^ antiporters, F_1_F_0_-type Na^+^-ATPase subunits, and K^+^ transporters with more than two-fold change at either 0.5 M or 1 M NaCl.

Gene	Accession No.	Function	Induction Factor	
			0.5 M NaCl	1 M NaCl
*nhaS3*	A0577	Na^+^/H^+^ antiporter localized in thylakoid membrane	‒–*^1^	2.4(± 0.1)
*atpA-II*	G0151	F_1_F_0_-type Na^+^-ATPase, subunit alpha	2.0 (± 0.3)	2.4(± 0.2)
*atpB-II*	G0148	F_1_F_0_-type Na^+^-ATPase, subunit A	2.5 (± 0.2)	1.3 (± 0.3)
*atpC-II*	G0145	F_1_F_0_-type Na^+^-ATPase, subunit epsilon	2.4 (± 0.3)	4.0 (± 0.2)
*atpD*	A0749	F_1_F_0_-type Na^+^-ATPase, subunit beta	2.5 (± 0.3)	2.9 (± 0.7)
*atpF-II*	G0150	F_1_F_0_-type Na^+^-ATPase, subunit B	2.0 (± 0.4)	2.1 (± 0.5)
*atpG-II*	G0152	F_1_F_0_-type Na^+^-ATPase, gamma subunit	2.3 (± 0.2)	‒–*^1^
*atpH-II*	G0149	F_1_F_0_-type Na^+^-ATPase, subunit C	1.5 (± 0.1)	2.1(± 0.0)
*kdpA*	G0060	K^+^-transporting ATPase, A subunit	32.0 (± 3.1)	2.0 (± 0.3)
*kdpB*	G0059	K^+^-transporting ATPase, B subunit	33.0 (± 4.7)	2.2 (± 0.3)
*kdpC*	G0055	K^+^-transporting ATPase, C subunit	13.9 (± 2.6)	1.3 (± 0.2)
*kdpD*	G0054	K^+^-transporting ATPase, D subunit	20.2 (± 6.5)	1.3 (± 0.1)
*kdpF*	G0057	K^+^-transporting ATPase, F subunit	20.6 (± 0.3)	1.5 (± 0.1)

*1, the value is not described because the induction factor is less than 1.0. Each value indicates the ratio of the level expression in stressed cells to that in control cells. Values shown are means (±SD) of three independent experiments.

**Table 2:** Gene expression related to compatible solute synthesis and glycogen synthesis with more than two-fold change at either 0.5 M or 1 M NaCl.

Gene	Accession No.	Function	Induction Factor	
			0.5 M NaCl	1 M NaCl
*glpD*	A2852	Glycerol-3-phosphate dehydrogenase	9.9 (± 1.1)	7.9 (± 1.0)
*ggpS*	A2851	Glucosylglycerol-3-phosphate synthase	9.3 (± 2.7)	6.9 (± 1.7)
*stpA*	A2841	Glucosylglycerol-3-phosphatase	2.6 (± 0.3)	1.5 (± 0.2)
*spsA*	A0888	Sucrose-phosphate synthase	2.5 (± 0.1)	2.6 (± 0.4)
*sppA*	A0887	Sucrose-phosphate phosphatase	2.1 (± 0.2)	2.0 (± 0.5)
*glgC*	A0095	Glycerol-3-phosphate dehydrogenase	−2.1 (± 0.2)	−2.4 (± 0.1)
*A2819*	A2819	1,4-Alpha-glucan branching enzyme	−2.1 (± 0.1)	−1.8 (± 0.0)
*glgP*	A2139	Alpha-glucan phosphorylase	2.1 (± 0.3)	2.2 (± 0.1)
*malQ*	A0330	4-Alpha-glucanotransferase	2.5 (± 0.4)	2.0 (± 0.4)

Each value indicates the ratio of the level expression in stressed cells to that in control cells. Values shown are means (±SD) of three independent experiments.

**Table 3:** Gene expression related to the Calvin cycle, the CCM, glycolysis, the ED pathway, and the OPP pathway with more than two-fold change at either 0.5 M or 1 M NaCl.

Gene	Accession No.	Function	Induction Factor	
			0.5 M NaCl	1 M NaCl
*rbcL*	A1798	Rubisco large subunit gene	−6.2 (± 1.9)	−5.9 (± 0.4)
*rbcS*	A1796	Rubisco small subunit	−5.5 (± 0.1)	−6.8 (± 0.3)
*rbcX*	A1797	Rubisco chaperone	−5.0 (± 0.3)	−6.3 (± 0.3)
*prk*	A2665	Phosphoribulokinase	−1.5 (± 0.2)	−2.5 (± 0.5)
*prk*	A2857	Phosphoribulokinase	−2.6 (± 0.4)	−3.5 (± 0.1)
*gap2*	A0106	Glyceraldehyde-3-phosphate dehydrogenase (Gapdh2)	−4.1 (± 0.6)	−2.8 (± 0.5)
*glpX*	A1301	Fructose-1,6-bisphosphatase/sedoheptulose 1,7-bisphosphatase	−4.0 (± 0.5)	−4.5 (± 1.6)
*ccmK*	A2613	Carboxysome shell protein (hexamer)	−3.4 (± 0.4)	−5.1 (± 2.3)
*ccmK*	A2612	Carboxysome shell protein (hexamer)	−1.9 (± 0.3)	−3.1 (± 0.4)
*ccmO*	A2389	Carboxysome shell protein (pseudohexamer)	2.4 (± 0.3)	1.1 (± 0.0)
*ccmL*	A1801	Carboxysome shell protein	−4.2 (± 0.3)	−4.8 (± 1.1)
*ccmM*	A1800	Carboxysome assembly protein	−2.7 (± 0.2)	−2.4 (± 0.2)
*pgm*	A0165	Phosphoglucomutase	−2.6 (± 0.4)	−2.4 (± 0.2)
*pgm*	A1492	Phosphoglucomutase	−1.1 (± 0.2)	2.3 (± 0.2)
*pfkA*	A0162	6-Phosphofructokinase	1.9 (± 0.0)	2.6 (± 0.0)
*fbp*	A0329	Fructose-1,6-bisphosphatase	2.4 (± 0.3)	‒–*^1^
*fba*	A1352	Fructose-1,6-bisphosphate aldolase class II	−2.3 (± 0.5)	−2.5 (± 0.2)
*fbaB*	A0010	Fructose-1,6-bisphosphate aldolase class I	3.4 (± 0.3)	1.7 (± 0.2)
*tpiA*	A0595	Triosephosphate isomerase	−1.2 (± 0.1)	−2.3 (± 0.1)
*gap1*	A2697	Glyceraldehyde-3-phosphate dehydrogenase (Gapdh1)	3.4 (± 0.7)	2.2 (± 0.4)
*pgk*	A1585	Phosphoglycerate kinase	−3.0 (± 0.3)	−2.4 (± 0.1)
*gpmB*	A2560	Phosphoglycerate mutase	−2.9 (± 0.1)	−2.4 (± 0.1)
*eno*	A0073	2-Phosphopyruvate hydratase	−2.8 (± 0.2)	−4.0 (± 0.6)
*zwf*	A1459	Glucose-6-phosphate 1-dehydrogenase	4.4 (± 0.4)	2.5 (± 0.2)
*edd*	A0652	6-Phosphogluconate dehydratase	−2.5 (± 0.3)	−2.4 (± 0.3)
*eda*	A0130	2-Keto-3-deoxygluconate-6-phosphate aldolase	3.6 (± 0.6)	3.7 (± 0.7)

*1, the value is not described because the induction factor is less than 1.0.

**Table 4:** Gene expression related to the TCA cycle, the urea cycle, and polyamine synthesis with more than two-fold change at either 0.5 M or 1 M NaCl.

Gene	Accession No.	Function	Induction Factor	
			0.5 M NaCl	1 M NaCl
*pyk*	A1658	Pyruvate kinase	−3.1 (± 0.4)	−2.6 (± 0.2)
*A0353*	A0353	Pyruvate dehydrogenase E1 alpha subunit	−3.4 (± 0.4)	−4.0 (± 0.2)
*pdhB*	A0655	Pyruvate dehydrogenase E1 beta subunit	−3.8 (± 0.5)	−3.7 (± 0.3)
*A0110*	A0110	Pyruvate dehydrogenase E2 subunit	−2.5 (± 0.2)	−3.5 (± 0.5)
*acnB*	A1683	Methylisocitrate dehydratase	−1.5 (± 0.1)	−3.0 (± 0.5)
*glnN*	A0246	Glutamine synthetase	−2.4 (± 0.2)	−3.0 (± 0.2)
*carB*	A2488	Carbamoyl-phosphate synthase large subunit	−2.4 (± 0.6)	−1.7 (± 0.2)
*A2770*	A2770	2-Ketoglutarate decarboxylase	2.2 (± 0.3)	2.7 (± 0.7)
*A2771*	A2771	Succinate-semialdehyde dehydrogenase	3.3 (± 0.3)	1.9 (± 0.1)
*sdhA*	A2569	Succinate dehydrogenase flavoprotein subunit	2.2 (± 0.2)	‒–*^1^
*argG*	A2806	Argininosuccinate synthase	−2.6 (± 0.3)	−2.3 (± 0.2)
*argH*	A2487	Argininosuccinate lyase	−1.6 (± 0.2)	−2.3 (± 0.1)
*speB*	A1109	Agmatinase	−3.4 (± 0.6)	−1.8 (± 0.1)
*speE*	A2283	Spermidine synthase	−5.5 (± 0.6)	−3.2 (± 0.3)
*metK*	A1714	S-adenosylmethionine (SAM) synthetase	−2.5 (± 0.3)	−2.9 (± 0.2)
*speH*	A0430	SAM decarboxylase	−10.2 (± 1.8)	−4.0 (± 0.1)

*1, the value is not described because the induction factor is less than 1.0.

**Table 5:** Gene expression of the photosynthesis reaction centers, phycobilisome, cytochrome *b_6_f*, electron carrier proteins, and ATP synthase with more than two-fold change at either 0.5 M or 1 M NaCl.

Gene	Accession No.	Function	Induction Factor	
			0.5 M NaCl	1 M NaCl
*psaA*	A1961	PSI P700 apoprotein A1	−2.2 (± 0.1)	−1.6 (± 0.0)
*psaB*	A1962	PSI P700 apoprotein A2	−2.3 (± 0.2)	−1.4 (± 0.0)
*psbA-II*	A0157	PSII D1 subunit	−3.3 (± 0.3)	−1.6 (± 0.3)
*psbA*	A1418	PSII D1 subunit	−3.5 (± 0.4)	−1.7 (± 0.3)
*psbA*	A2164	PSII D1 subunit	1.7 (± 0.2)	3.5 (± 1.7)
*psbD*	A1560	PSII D2 subunit	−2.1 (± 0.1)	−1.8 (± 0.0)
*apcA*	A1930	Allophycocyanin (APC) subunit alpha	−4.8 (± 0.2)	−3.3 (± 0.6)
*apcB*	A1929	APC subunit beta	−4.0 (± 0.6)	−3.6 (± 0.6)
*apcE*	A2009	PBS linker protein	−2.5 (± 0.2)	−1.5 (± 0.1)
*cpcA*	A2210	Phycocyanin (PC) subunit alpha	−4.6 (± 0.2)	−2.6 (± 0.2)
*cpcB*	A2209	PC subunit beta	−4.4 (± 0.3)	−2.6 (± 0.6)
*cpcE*	A2213	PC alpha subunit phycocyanobilin lyase	‒–*^1^	3.0 (± 0.4)
*cpcF*	A2214	PC subunit alpha phycocyanobilin lyase	1.2 (± 0.1)	3.8 (± 0.2)
*petA*	A1910	Cytochrome *f*	−3.5 (± 0.4)	−5.8 (± 1.5)
*petB*	A0842	Cytochrome *b_6_*	−2.4 (± 0.1)	−3.1 (± 0.1)
*petF*	A2325	Ferredoxin	−3.4 (± 0.6)	−3.7 (± 0.4)
*petH*	A0853	Ferredoxin-NADP reductase	−2.9 (± 0.3)	−4.5 (± 0.4)
*atpA*	A0734	F_1_F_0_ ATP synthase subunit alpha	−3.5 (± 0.8)	−3.8 (± 0.5)
*atpB*	A0739	F_1_F_0_ ATP synthase subunit A	−3.3 (± 0.3)	−11.6 (± 3.1)

*1, the value is not described because the induction factor is less than 1.0.

## Supplementary Materials

The following are available online at https://www.mdpi.com/2218-1989/9/12/297/s1. Table S1: Gene expression of the photosynthesis reaction center, phycobilisome, cytochrome *b_6_f*, and electron carrier proteins with less than two-fold induction at either 0.5 M or 1 M NaCl, except for the genes shown in Table 5.
