# Supplementary material for: Short-Term Temporal Metabolic Behavior in Halophilic Cyanobacterium Synechococcus sp. Strain PCC 7002 after Salt Shock
Source: Metabolites. 2019 Dec 5;9(12):297. doi: 10.3390/metabo9120297 (PMC6950573; doi:10.3390/metabo9120297)
Supplement: Supplementary file 1 [file metabolites-09-00297-s001.zip › Supplemental Table S1.pdf]

Table S1: Gene expression of the photosynthesis reaction center, phycobilisome, cytochrome *b6f*, and electron carrier proteins with more than 2-fold changes at either 0.5 M or 1 M NaCl, except for the genes shown in Table 5.

Gene	Accession No.	Function	Induction factor	
			0.5 M NaCl	1 M NaCl
psaC	A1589	PSI iron–sulfur center subunit VII	–2.3 (±0.4)	−1.1(±0.0)
psaD	A0682	PSI subunit II	−2.6(±0.3)	−1.7(±0.4)
psaE	A1393	PSI reaction center subunit IV	−3.1 (±0.7)	−4.5 (±0.3)
psaF	A1008	PSI reaction center subunit III	−3.4 (±0.5)	−4.6 (±2.6)
psaK	A2401	PSI reaction center subunit X	−3.0 (±0.2)	−2.3 (±0.2)
psaL	A2620	PSI reaction center subunit XI	−2.3 (±0.1)	−1.9 (±0.1)
ycf3	A1566	PSI assembly protein	−2.6 (±0.7)	−4.4 (±0.5)
ycf4	A0229	PSI assembly protein	−2.3 (±0.3)	−2.2 (±0.1)
psbB	A1759	PSII 47 kDa protein	−3.3 (±0.3)	−1.9(±0.3)
psbC	A1559	PSII 44 kDa protein	−2.4 (±0.3)	−2.3 (±0.1)
psbD	A1560	PSII subunit D2	−1.9 (±0.1)	–2.1 (±0.1)
psbH	A0808	PSII subunit H	−3.1 (±0.1)	−3.1(±0.3)
psbO	A0269	PSII manganese–stabilizing protein	−3.8 (±0.2)	−3.4 (±0.5)
psbU	A0322	PSII 12 KDa protein	−4.7 (±0.5)	−2.7(±0.4)
psbW	A1258	PSII 13 KDa protein	−1.2 (±0.1)	−2.4 (±0.2)
psbZ	A2533	PSII subunit Z	−2.4 (±0.5)	−4.4 (±0.8)
apcC	A1928	APC–associated PBS 7.8 kDa core–linker polypeptide	−4.2 (±0.0)	−3.5 (±0.2)
apcF	A1631	APC subunit beta–18	−3.6 (±0.3)	−1.9 (±0.3)
cpcC	A2211	PC–associated rod linker protein	−4.6 (±0.4)	−1.6 (±0.5)
cpcD	A2212	PC–associated rod terminated linker protein	−3.9 (±0.8)	−1.1 (±0.1)
cpcG	A0639	PBS rod–core linker polypeptide	−4.3 (±0.5)	−2.5 (±0.5)
petD	A0841	Cytochrome *b*$_6$/*f* subunit IV	−2.2 (±0.2)	−4.4 (±0.7)

petC	A1909	Cytochrome b_6/f iron–sulfur subunit	−3.6 (±0.6)	−7.3 (±1.6)
petF	A2192	Ferredoxin 2Fe–2S	−1.4 (±0.2)	−2.8 (±0.3)
petF	A2326	Ferredoxin I (2Fe–2S)	−1.8 (±0.3)	−2.9 (±0.4)
petH	A0853	Ferredoxin–NADP reductase	−2.9 (±0.5)	−4.5 (±0.4)
petJ	A0167	Cytochrome c_6	−2.3 (±0.2)	−2.2 (±0.1)
atpC	A0750	ATP synthase F1, epsilon subunit	−3.7 (±0.2)	−4.5 (±0.5)
atpD	A0749	ATP synthase beta chain	−2.5 (±0.3)	−2.9 (±0.7)
atpE	A0738	ATP synthase C chain	−4.0 (±0.1)	−5.9 (±0.5)
atpF	A0736	ATP synthase B chain	−4.2 (±0.5)	−4.8 (±0.6)
atpG	A0737	ATP synthase B chain	−3.6 (±0.3)	−4.8 (±0.4)
atpG	A0733	ATP synthase F1, gamma subunit	−3.6 (±0.6)	−3.9 (±0.3)
atpH	A0735	ATP synthase F1, delta subunit	−4.3 (±0.4)	−3.9 (±0.2)

Each value indicates the ratio of the level expression in stressed cells to that in control cells. Values shown are means (±SD) of results of three independent experiments.
